# Heats of Solution, Transition, and Formation of Three Crystalline Forms of Metaboric Acid

**DOI:** 10.6028/jres.068A.012

**Published:** 1964-02-01

**Authors:** Marthada V. Kilday, Edward J. Prosen

## Abstract

The three crystalline forms of metaboric acid HBO_2_ were prepared, purified, and analyzed. Heats of solution in water or of reaction with sodium hydroxide solution were compared with those of orthoboric acid H_3_BO_3_(c). The best values for the heats of transition at 25 °C are: (c,I) to (c,II), 2.33±0.23 kcal/mole; (c,II) to (c,III), 1.30±0.05 kcal/mole; (c,I) to (c,III), 3.63±0.24 kcal/mole. The following heats of formation at 25 °C were derived: −192.77 ± 0.35 kcal/mole for the cubic HBO_2_(c,I), −190.43 ±0.34 kcal/mole for the monoclinic HBO_2_ (c,II), and −189.13 ± 0.34 kcal/mole for the orthorhombic HBO_2_(c,III).

## 1. Introduction

Three crystalline forms of metaboric acid HBO_2_ were identified and described by Kracek, Morey, and Merwin [1]^1^ in 1938. The three forms are the cubic form HBO_2_(c,I), the monoclinic form HBO_2_(c,II), and the orthorhombic form HBO_2_(c,III). Although heat-of-solution data [2–6] and other thermodynamic data are available on the monoclinic and orthorhombic forms, no heat of solution or formation data and little other thermodynamic information other than that of Kracek, Morey, and Merwin are available on the most stable form, the cubic. (See, however, the preliminary report of part of the present work [7].) An investigation [18] of the equilibrium decomposition pressures of orthoboric acid into water vapor and each of the three metaboric acids yielded both heat-of-formation [(c,I), −192.6; (c,II), −190.6; (c,III), −189.0 kcal/mole] and entropy values for each of the forms. However, since the uncertainty in the heat of formation derived from those measurements was large, it was desirable to determine the heats of formation (or differences in heat of formation) by a more direct means.

The three crystalline forms of metaboric acid were prepared, purified, and analyzed. Heats of hydrolysis in water and heats of neutralization in sodium hydroxide solution were measured and compared with similar heats using orthoboric acid. From these heats of solution or neutralization, heats of transition and formation of the three forms of metaboric acid may be derived, since, as indicated by the work of Hibben [8], H_3_BO_3_ and HBO_2_ form the same final products in solution.

## 2. Preparation and Analyses of Samples

The experiences of Kracek [1] and Tazaki [9] as well as other thermodynamic and mechanism studies [18] of the decomposition pressures of orthoboric acid were considered in the development of the methods of preparation of the metaboric acid samples described in this paper.

In general the samples were identified by X-ray diffraction,^2^ refractive index,^3^ and density measurements which were in agreement with the work of Kracek [1] and Tazaki [9]. The boron content of the various materials was determined from titrations (in the presence of an excess of d-mannitol) of aqueous solutions of weighed samples using 0.1*N* sodium hydroxide and a Beckman *p*H meter with a glass electrode and a calomel reference electrode.

Quick identification and separation of the various crystals were made by flotation in the liquids listed in table 1 which also lists the densities of the metaboric acids and orthoboric acid.

For the sake of brevity in this paper HBO_2_(c,I), HBO_2_(c,II), and HBO_2_(c,III) will sometimes be referred to as (c,I), (c,II), and (c,III), respectively.

### 2.1. H_3_BO_3_(c)

Orthoboric acid, analytical grade reagent, was recrystallized three times from aqueous solution and air-dried at room temperature. It was stored in a desiccator containing a saturated solution of calcium bromide which maintains a relative humidity of about 20 percent at room temperature. A spectroscopic analysis^4^ of a sample prepared in this manner indicated a total impurity of not more than 0.001 mole percent. Titration analyses of the final calorimetric solutions of boric acids in water indicated a weight of boric acid averaging 99.72 ± 0.09 (sdm) percent of the sample weight; the accuracy of the titration analyses was estimated to be a few tenths of a percent the total boric acid.

### 2.2. HBO_2_(c,III)

It should be possible in preparing the metabolic acids from orthoboric acid to choose conditions of temperature, pressure, and humidity which would result in pure products if the conditions were accurately controlled. However, the preparations described here contained mixtures of the metaboric acids and orthoboric acid which were separated by flotation methods and/or analyzed to determine the composition of the sample.

**Figure 1:**
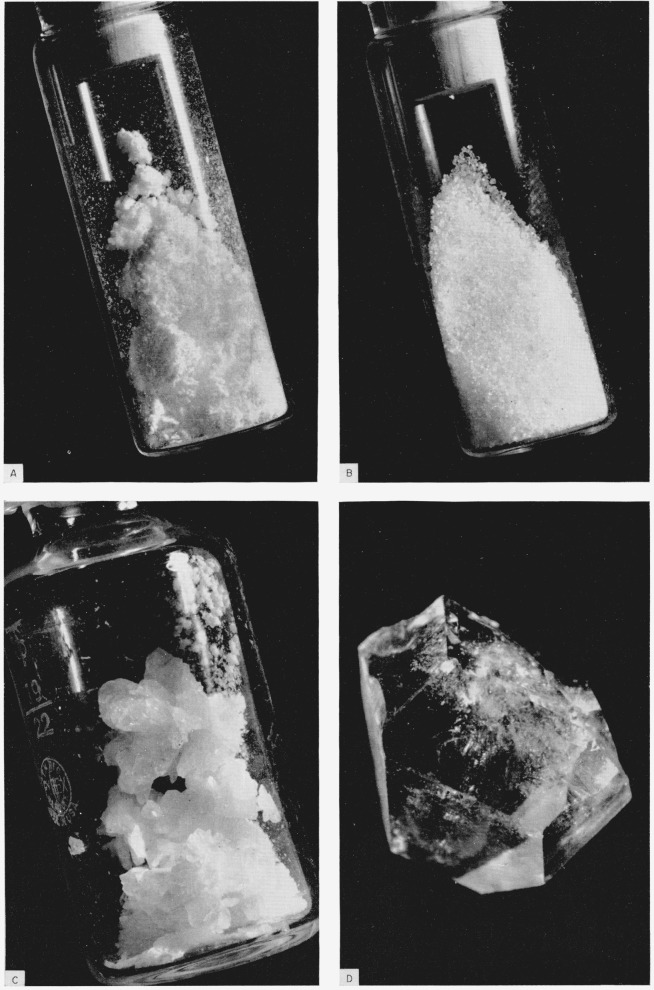
The various crystal forms of metaboric acid: A. orthorhombic, B. cubic, C. monoclinic, and D. a large single cyrstal of the cubic *HBO_2_(c,I*), actual size, approximately 3.5 mm; A, B, and C are approximately actual size.

The orthorhombic form of metaboric acid was obtained by dehydration of orthoboric acid in open Petri dishes at 65 °C and the samples were weighed every 2 or 3 days during the dehydration. The relative humidity of the atmosphere generally ranged from 15 to 30 percent but occasionally for brief periods it was as high as 55 percent. The theoretical weight for metaboric acid was reached in 1 to 3 weeks, however, this was not an exact indication of the completeness of dehydration since orthoboric acid volatilizes to an appreciable extent at 65 °C. At this temperature and humidity the only impurity expected and found in the product was orthoboric acid, the amount of which varied somewhat with the atmospheric conditions during preparation. The products generally contained 80 to 90 percent (c, III). The samples were stored in glass-stoppered bottles over anhydrous magnesium perchlorate drying agent, and subsequent manipulations were performed in a dry box. The appearance of the product may be seen in figure 1a.

### 2.3. HBO_2_(c,II)

The monoclinic form of metaboric acid was prepared at 120 °C by dehydration of wet orthoboric acid in bottles with loose-fitting glass stoppers to impede the escape of water vapor. The dehydration generally required 3 to 4 weeks. Extended heating was not desirable because greater contamination with (c,I) developed. Formation of (c,II) was apparent from the presence of globules or treelike structures. The best yield of the (c,II) was obtained when the starting material was orthoboric acid which had not been thoroughly dried but was still moist with the liquid from recrystallization.

These preparations contained the three crystal forms of metaboric acid as well as some orthoboric acid. The purification and all subsequent manipulations of the (c,II) were performed in a dry box to prevent the sample from reacting with atmospheric moisture. The product was first washed thoroughly with spectroscopic-grade (H_2_O-free) carbon tetrachloride to remove H_3_BO_3_(c) and (c,III) by flotation and solution. Figure 1c shows the material at this stage of the purification. The remaining clusters of crystals were crushed (but not ground) in a mortar. When they were added to ethyl iodide (analytical reagent), the (c,II) and (c,I) sank to the bottom. The floating crystals and the ethyl iodide were removed before adding ethylene bromide in which (c, II) floats but (c,I) does not. The floating crystals, mostly needlelike in form were removed, washed with H_2_O-free CCl_4_, and dried at 120 °C for about 30 min before storing in a glass-stoppered bottle over anhydrous magnesium perchlorate.

Flotation did not completely separate (c,T) from (c,II) due to air-bubble effects and to inclusion of (c,I) by (c,II). However, (c,II) dissolves moderately rapidly in water at 25 °C while (c,I) is only very slightly soluble at that temperature, thus it was possible to determine the amount of (c,II) by titration of an aqueous solution of the sample. About 1 percent of (c,I) was found in the best samples; details will be given in the analyses of the calorimetric solutions.

### 2.4. HBO_2_(c,I)

The most stable form of metaboric acid, the cubic HBO_2_(c,I), has been prepared by several methods. One method was similar to the preparation of (c,II) except that the orthoboric acid was not wet initially and a period of 4 to 5 weeks was required to complete the dehydration. This product consisted of crystals of (c,I) approximately 1 mm in size mixed with some (c,II). Figure 1b shows this product after purification.

Another method of preparing the (c,I) resulted in a product which contained essentially no impurities. Thin glass spherical bulbs, 1.5 cm diam, were blown at the end of 4 mm (o.d.) glass tubing; the overall length was approximately 12 cm. The bulbs were filled with orthoboric acid and weighed. After heating at 110 °C for approximately 4 weeks the samples had reached constant weight at approximately that of metaboric acid.

A third method produced crystals which were generally smaller than those formed in the other two methods, but occasionally larger crystals were formed; the largest single crystal obtained was approximately 3.5 mm in size and is shown in figure 1d. In this method 5 g of orthoboric acid, 15 g of (c,III), and a few seed crystals of (c,I) were sealed, after brief evacuation, in a 100-ml Pyrex-glass ampoule and heated at 180 °C. Sometimes in a day or two, the melt developed “cloudiness” indicating crystal formation, but at other times it was as much as a week before crystallization was visible; 2 to 5 weeks was allowed for crystal aggregation but this was no guarantee of large crystal formation. As the ampoule cooled, the mixture formed a solid mass which adhered tightly to the surface of the glass but apparently did not attack the glass, because it was possible after soaking with water to scrape off the crystals leaving a smooth glass surface.

Samples of (c,I) of high purity were obtained by washing the products of the above preparations with water since the cubic form is only very slightly soluble. The cubic crystal surfaces were attacked after several hours exposure to water at room temperature, but extended washing with methanol effectively dissolved the other forms of HBO_2_ and did not mar the crystal surfaces. We feel that a sample of high purity was obtained in this way although it was not possible to obtain a quantitative analysis with respect to the crystal form. When weighed samples were dissolved in boiling water and titrated in the presence of excess d-mannitol, 99.89 ± 0.03 percent of the theoretical amount of boric acid was found in the analyses of four samples.

## 3. Apparatus and Procedures

### 3.1. Calorimetric Apparatus and Procedures

**Figure 2:**
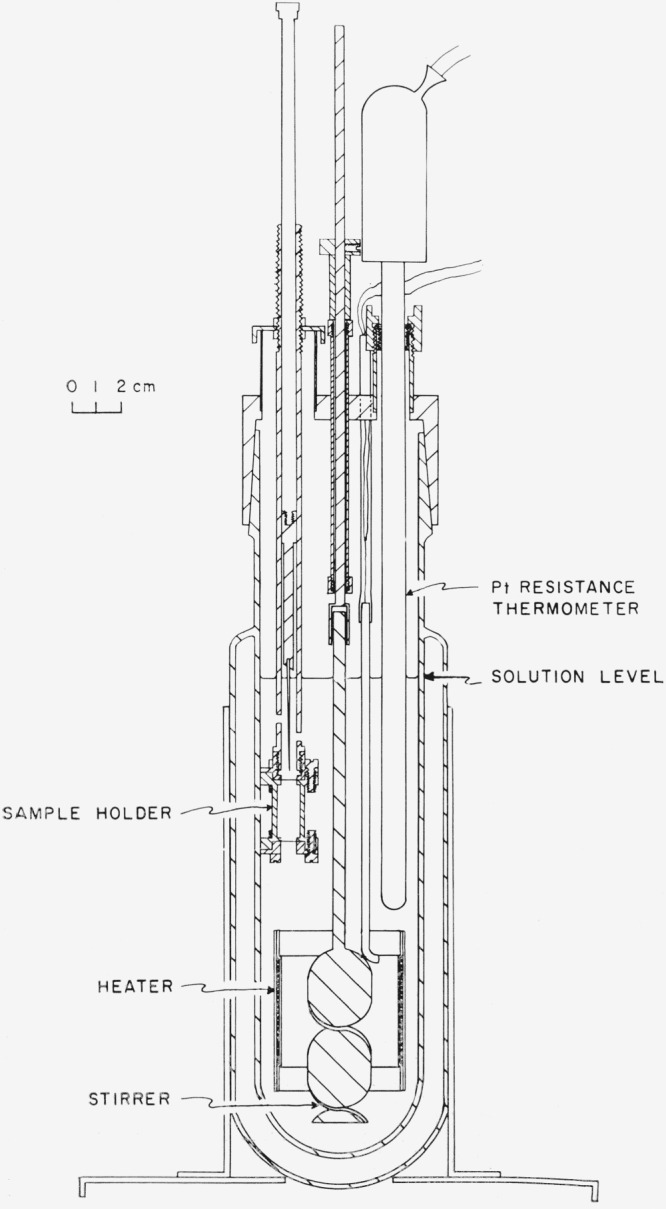
The solution calorimeter.

The calorimeter employed in these experiments is shown in figure 2 and described in [10]. The temperature of the water bath was maintained at approximately 25 °C or 40 °C and normally varied by ±0.003 °C during an experiment. The bath, temperature controls, and method and instruments for measuring time, temperature, and electrical energy have been described previously [10, 11, 12].

Each calorimetric experiment included a preliminary equilibration period of 20 to 30 min, a 20-min initial rating period, an electrical calibration period, a 20-min middle rating period, a chemical reaction period, and a 20-min final rating period. The temperature was recorded at 2-min intervals during the rating periods, and at 1-min intervals during the calibration and chemical reaction periods. The electrical calibration period lasted either 20 min with 7 or 9 min of heating or 10 min with 5 min of heating; temperature rises were 0.01 ohm per minute of heating. The chemical reaction periods generally made 40 min long for the solution in water and 20 or 30 min long for the solution in sodium hydroxide to insure complete solution.

In early experiments glass bulbs were used to contain the samples with a holder similar to that described in [12]. In later experiments the Monel capsule shown in figure 2 was used and platinum disks, 1 mil thick, closed the ends of the sample holder. The stirring rate in early experiments was 500 rpm but was later increased to 1000 rpm in order to prevent the (c,I) from settling to the bottom, and to dissolve the samples within a reasonable length of time for calorimetric measurements.

Three series of calorimetric experiments to be reported differ somewhat in procedure and in the composition of the calorimetric systems.

#### Series I

In the earlier experiments, the samples were contained in glass bulbs and the stirring rate of 500 rpm was used. The heats of solution in water were measured using 500 g (27.8 moles) of water in the calorimeter. For measuring the heat of reaction in 2*N* sodium hydroxide solution, the calorimetric solution was prepared in the calorimeter by adding 135 g of a stock solution of 10*N* sodium hydroxide to 402 g (22.3 moles) of water. In this series, heats of solution in water and in 2*N* sodium hydroxide were measured at 25 °C for orthoboric acid and metaboric acid (c,II), and heats of solution in 2*N* sodium hydroxide were measured at 40 °C for orthoboric acid and metaboric acids (c, II) and (c,I).

The process of filling the thin glass bulbs with the samples of the metaboric acids was a tedious and time-consuming one, and the evacuation and sealing of the bulbs after filling introduced the possibility of decomposition of the samples. These difficulties were eliminated in later experiments where the Monel capsule replaced the glass bulb as the sample container. The containers for the metaboric acids were always filled in a dry box but those for orthoboric acid were filled in the room atmosphere.

#### Series II

These experiments were similar to those of Series III except that the samples were enclosed in glass bulbs rather than in the Monel capsule.

#### Series III

In these experiments, using the Monel capsule and the 1000-rpm stirring rate, the heat capacity of the system was reduced. For the heats of solution in water the calorimeter contained 452 g (25 moles) of water. For the heats of reaction in 2*N* sodium hydroxide the calorimeter contained 362 g (20 moles) of water plus 122 g of the stock solution of 10*N* sodium hydroxide. In this series heats of solution in water were measured at 25 °C for orthoboric acid, metaboric acids (c,III) and (c,II); and heats of reaction in 2*N* sodium hydroxide at 25 °C were measured for orthoboric acid, metaboric acids (c, III), (c,II), and (c,I).

The cubic HBO_2_(c,I) is the most stable form of metaboric acid and the solution in which the (c,I) dissolved completely within a reasonable length of time and under conditions suitable for calorimetric measurements was also used for measurements of the heats of solution of the two less stable forms of metaboric acid and of orthoboric acid. Solution of (c,I) was accomplished in a 2*N* sodium hydroxide solution at 40 °C in approximately 30 min at a stirring rate of 500 rpm when the sample was crushed to pass a No. 200 standard sieve. The crushing and sieving of the sample was a tedious operation which was performed in the room atmosphere; the gain in weight resulting from reaction with and/or adsorption of moisture from the atmosphere amounted to less than 1 percent of the sample weight. No difference was detected by X-ray analysis between the sieved and the uncrushed samples.

In preparing the calorimetric samples of (e,I), the freshly sieved material was heated at 120 °C for 1 hr and cooled in a desiccator. Thin glass bulbs were filled with the sample in a dry box; the bulbs were then removed from the dry box, attached to a vacuum manifold, and sealed off under reduced pressure. These samples were used for solution in sodium hydroxide at 40 °C. It was later learned that the sample could also be dissolved at 25 °C in approximately 50 min by doubling the stirring rate. In the two experiments under these conditions the Monel capsule was used as the sample container rather than the glass bulbs; the capsule was filled in a dry box.

### 3.2. Determination of Energy for Initiating Reactions

The energy for breaking the glass bulbs was too small to be measured in this system; the limit of detection was about 0.4 j. Other investigators [14] have measured the energy and found that 0.2 j or less was required to break thin glass bulbs. When the Monel capsule was used to contain the sample, the energy required to break the two platinum disks was affected by several factors other than the properties of the disks, namely, the reaction rate, the particle size, and the extent to which the capsule was filled. Thus there was a large percentage of uncertainty in the determination of this energy.

The disk-breaking energy was determined as follows. In two experiments where the capsule contained only water the energy for breaking the the disks was found to be approximately 1.5 j/expt.; this was near the limit of detection in this calorimeter, and not typical of the energies involved in the metaboric acid experiments because there was no friction resulting from the presence of crystalline samples. It was believed that disk-breaking energies more comparable to those of the experiments reported later in this paper were obtained in three series of experiments in which the heats of solution in water at 25°C were measured for NaCl and KCl. The disk-breaking energy was obtained from the difference between the measured energy and the theoretical energy calculated from the weight of sample, using the following heats of solution: 1.001 kcal/mole for NaCl (dilution = 1000 H_2_O) [15] and 4.187 kcal/mole for KCl (dilution = 1500 H_2_O) [15]. The average disk-breaking energies obtained were, respectively, 3.86±0.24 (sdm) j/expt. and 9.84±1.01 (sdm) j/expt. The mean of these values, 7±4 j/expt., was used to correct the experiments of series III.

### 3.3. Thermal Equilibrium

A disadvantage in the use of a glass, vacuum-jacketed calorimeter is that thermal equilibrium is reached slowly between the constant-temperature bath, the glass walls of the calorimeter, and the calorimeter solution after a change in the temperature of the calorimeter solution. In order to determine the magnitude of errors resulting from failure to acheive thermal equilibrium in both exothermic and endothermic reactions, an experiment was performed which involved no chemical energy. An increase in temperature was produced by electrical heating; a decrease, by inserting a copper rod precooled in liquid nitrogen into a copper tube which extended into the calorimeter solution and withdrawing the rod when the desired temperature drop was acheived. A 2-hr equilibration period was followed by a 50-min initial rating period, a 10-min heating period, which included 5 min of electrical heating, a 60-min middle rating period, a 10-min cooling period during which the copper rod was inserted for 1½ min, and a 60-min final rating period. The results indicated that equilibrium was not reached until at least 45 min after the cessation of electrical heating or withdrawal of the cold rod. It was estimated that an error of 0.5 percent for a temperature rise of 0.05 ohm resulted from using reaction periods of only 10 or 20 min (as in the calibrations), and an error of 1.0 percent for a temperature drop of 0.02 ohm. For the experiments reported in this paper, the largest errors (1 to 2 percent of the total energy absorbed by the system) occurred in the aqueous solutions which were endothermic. The errors in the exothermic reactions (solutions in sodium hydroxide) were about 0.5 percent or less. Although these errors were considered in the overall uncertainties, they tend to cancel in the calculation of the heats of formation and transition.

### 3.4. Chemical Analyses and Procedure

The actual composition in terms of species of the final calorimetric solutions was not determined. However, Hibben [8] found from infrared and Raman measurements on aqueous solutions of boric acid and borates that only H_3_BO_3_ and BO_2_^−^ are present in dilute solutions. Thus, when ortho-or metaboric acid is dissolved in water, H_3_BO_3_(aq) is the product; and, when dissolved in aqueous bases, the metaborate ion is formed. By assuming that the products of solution are the same for orthoboric acid and for the three metaboric acids, the heat of formation for any of the metaboric acids can be calculated knowing only the heat of solution of orthoboric acid and of the metaboric acid, and the heats of formation of water and of orthoboric acid.

The final calorimetric solutions in water were analyzed by titration for orthoboric acid, but it was not possible to obtain an accurate analysis of the solutions in the sodium hydroxide. Therefore, these heats of reaction are based on the number of moles calculated from the sample weight and the average composition of the samples obtained from analyses of the aqueous solutions. The final calorimetric solution from the water experiments was diluted to 500 ml or 1000 ml and appropriate aliquots were taken for titration in the presence of excess d-mannitol with a standard 0.1*N* sodium hydroxide solution. A Beckman *p*H meter was used to indicate the end points which occurred at about *p*H 7.8. These titrations are probably accurate to a few tenths of a percent. The ratio of the amount of boric acid found in the titrations to the amount of boric acid based on the sample weight is an indication of the purity of the sample with respect to chemical composition but not to crystal form. From the boric acid ratio it was possible to calculate the amount of orthoboric acid present in the samples of HBO_2_(c,III), and the average composition of these samples was applied in corrections calculated for the solutions of the same sample in sodium hydroxide where it was not possible to obtain an accurate analysis of the final calorimetric solutions.

The final calorimetric solutions of the samples of metaboric acid (c,II) in water contained the undissolved (c,I). Therefore, the final solution was filtered through a coarse filter paper and the filtrate was titrated as described above; the number of moles of boric acid found in this titration was assumed to be equal to the number of moles of (c,II) in the sample. the difference between this and the number of moles of metaboric acid in the sample as calculated from its weight, was taken as the amount of (c,I) in the sample. An approximate check on this value was obtained from titration of an aqueous solution of the undissolved residue. This value was not as accurate as that obtained from the weight of sample because the amount of (c,I) was only about 1 to 5 percent of the sample, and losses during the manipulations of such small crystals were likely to occur.

The approximate amount of (c,I) which dissolved in water at 25 °C was determined by adding a weighed sample of (c,I) to 452 ml of distilled water in the calorimeter and stirring at about 1000 rpm for 2 hr. The solution was then filtered through a fine filter paper and the filtrate was titrated for boric acid. As would be expected, more rapid solution occurred when the samples were of small particle size. Approximately 9 mg dissolved from a 0.3-g sample which was 40–70 mesh, 15 mg dissolved from a similar sample which was less than 100 mesh, and 2 mg dissolved from a 0.030 g sample (<100 mesh). The time during which the samples were exposed to the water in these experiments was about twice that in the calorimetric experiments; therefore, the solution of not more than 0.2 mg of (c,I) (or 0.02% of *S*) would be expected in the experiments given in table 3. The acutal amount of (c,I) which dissolved in water was so small and uncertain because of particle size that no correction was made for it.

Solutions prepared in the same manner as the calorimetric solutions of sodium hydroxide were analyzed by titration against weighed samples of potassium acid phthalate (NBS standard sample No. 84) using phenolphthalein indicator. These analyses were only approximations since no special precautions were used to exclude carbon dioxide from the calorimetric solutions. An analysis of a solution prepared from stock soln No. 1 [used in reactions of (c,I) at 40 °C] indicated a normality of 1.94; a solution prepared from stock soln No. 3 [used in reactions of H_3_BO_3_(c) at 25 °C] was found to be 2.27*N*; and one prepared from stock soln No. 4 [used in reactions of H_3_BO_3_ at 40 °C, and (c,II) at 40 °C and 25 °C] was 1.88*N*. Two stock solutions, A and B, were used alternately in the preparation of calorimetric solutions for the experiments in Series III. Initial analysis of a solution prepared from stock soln A indicated a normality of 1.79, and from stock soln B, 1.96; no change was observed in the final analyses of similar solutions.

## 4. Units and Physical Constants

The unit of energy for the calorimetric experiments reported in this paper is the absolute joule. For conversion to the thermochemical calorie, 1 thermochemical calorie = 4.1840 j.

Values for atomic weights were taken from the 1961 Table of Atomic Weights [16].

The masses of all samples given in the following tables have been corrected to the weights in vacuo using buoyancy factors calculated from densities given by Kracek, Morey, and Merwin [1].

## 5. Experimental Results

The heats of formation of the three metaboric acids were calculated from the heats of reaction of the metaboric acids and of orthoboric acid in water and in solutions of sodium hydroxide. The results of the experiments where these heats of reaction were determined are given in tables 2 through 6. Tables 2 through 5 give details of experiments on which our “best values” are based, and in table 6 the average values of 4 to 6 experiments for all other reactions are given.

In all experiments *S* is the weight (in vacuo) of sample in grams; *E_a_* is the energy equivalent of the initial system as determined from the electrical calibration which preceded each chemical reaction experiment; Δ*Rc* is the corrected temperature rise of the system [13]; *Q*, the product of *E_a_* and Δ*Rc*, is the total energy absorbed by the system; and Δ*H*, the enthalpy of reaction, is the heat absorbed (−*Q*, less any applicable corrections, ***−****q*) per mole of orthoboric acid or metaboric acid calculated from the weight of sample or determined from the number of moles of orthoboric acid found in the titrations of the final calorimetric solutions, H_3_BO_3_ titr. The overall uncertainty includes twice the experimental standard deviation of the mean and other uncertainties discussed in section 5.1. The H_3_BO_3_ ratio is the ratio of the number of moles of orthoboric acid found in titrations of the final calorimetric solutions to the number of moles of orthoboric acid calculated from the sample weight; *q_d_* is the correction for the energy required to break the platinum disks used in Series III.

The heats of solution of orthoboric acid in water at 25 °C and in sodium hydroxide solution at 25 °C and 40 °C are used in the calculations of the heats of formation of the three metaboric acids. The heat of solution in water at 25 °C has been determined by several investigators [2–5, 10, 19–25] and their values at a dilution of 1000 H_2_0 range from 5.17 kcal/mole to 5.56 kcal/mole; the more recent values are grouped between 5.24 and 5.28 kcal/mole.

We have measured the heat of solution of orthoboric acid in water in two groups of experiments and the results are given in table 6, groups 8 and 9 for Series II and Series III which are described in section 3.1. The samples in Series II were enclosed in glass bulbs which had been sealed after pumping for several hours at 10^−5^mm Hg pressure at room temperature; the samples in Series III were enclosed in the Monel capsule. The reaction periods for the experiments in group 8 were of 20-min duration while those in group 9 were of 40-min duration, thus the latter should be nearer thermal equilibrium at the end of the reaction period.

According to Smisko and Mason [19] the heat of solution of orthoboric acid in water does not change significantly between the dilutions of 500 H_2_O and 5000 H_2_O. Therefore no corrections for dilutions of H_3_BO_3_ are made in this paper since all values fall within the range of 900 to 2700 H_2_O.

The data for the measurements of the heats of solution of orthoboric acid in sodium hydroxide solution at 40 °C and at 25 °C are given in table 6, groups 1, 4, and 5. The largest uncertainty considered for these reactions was the possible variation in the concentration and/or composition of the calorimetric solutions of sodium hydroxide.

Our best value for the heat of transition from (c,III) to (c,II) is derived from the heats of solution in water at 25 °C (Series III) given in tables 2 and 3, and group 9 of table 6. The equation representing the mean heat of solution given in table 2 is

$$\begin{array}{l} {\text{HBO}}_{2}(c,\text{III})+1501\;H_{2}O(\text{liq})\to H_{3}{\text{BO}}_{3}(1500\;H_{2}O)\\ \;\Delta H(25\;{}^{\circ}C)=1.869\pm 0.187\;\text{kj}/\text{mole}\\ \;=0.447\pm 0.045\;\text{kcal}/\text{mole} \end{array}$$ (1)

and that for table 3 is

$$\begin{array}{l} {\text{HBO}}_{2}(c,\text{II})+901\;H_{2}O(\text{liq})\to H_{3}{\text{BO}}_{3}(900\;H_{2}O)\\ \;\Delta H(25\;{}^{\circ}C)=7.33\pm 0.22\;\text{kj}/\text{mole}\\ \;=1.752\pm 0.053\;\text{kcal}/\text{mole} \end{array}$$ (2)

The experimental uncertainty, 2(sdm), is large for eq (1) because the amount of heat absorbed during the reaction was small. The uncertainty with respect to sample composition also made a relatively large contribution to the overall uncertainty for eq (2).

The heat of reaction in table 2 is based on the number of moles of (c,III) calculated from the stoichiometric relationship between the weight of sample and the amount of boric acid found in titrations of the final calorimetric solutions. The corrections for the energy absorbed during the solution of the orthoboric acid were calculated from the results of group 9, table 6. The composition of the samples in group 7, table 6 was assumed to be the same as the average of those in table 2.

In table 3, the H_3_BO_3_ ratio is the ratio of the sum of the number of moles of (c,II) and of (c,I) titrated, to the number of moles of HBO_2_ calculated from the sample weight. From this it can be seen that only a few tenths of 1 percent of the sample weight was not accounted for in the titration analyses and this is within the limits of accuracy expected of the analytical methods employed. The extra care taken in the purification of this sample is evidenced by the fact that the amount of (c,I) impurity is much smaller and the composition of the samples is more uniform than those of the experiments in Series I given in group 10 of table 6, on which the average composition, 6 percent of (c,I), of the samples in groups 2 and 6 was based. The amount of (c,I) dissolved during the latter experiments was determined from the difference between the amount assumed to be present initially and the amount found in the undissolved residue at the end of the experiment. A correction for the heat of reaction of the (c,I) which dissolved, *q*_(c, I)_, was made using the results obtained in group 3 of table 6 and in table 5. A large uncertainty with respect to composition of sample was considered in the results of groups 2, 6, and 10; groups 2 and 6 had an additional uncertainty in the concentration of sodium hydroxide solutions.

The results given in tables 4 and 5 were used with the results of group 5, table 6, to calculate our best value for the heat of transition from (c,II) to (c,I). The mean heat of reaction for the experiments in table 4 is given by the equation,

$$\begin{array}{l} {\text{HBO}}_{2}(c,\text{II})+\text{NaOH}(\text{aq})\to [{\text{NaBO}}_{2}+H_{2}O](\text{soln})\\ \;\Delta H(25\;{}^{\circ}C)=-37.10\pm 0.74\;\text{kj}/\text{mole}\\ \;=-8.87\pm 0.18\;\text{kcal}/\text{mole} \end{array}$$ (3)

and for those in table 5,

$$\begin{array}{l} {\text{HBO}}_{2}(c,I)+\text{NaOH}(\text{aq})\to [{\text{NaBO}}_{2}+H_{2}O](\text{soln})\\ \;\Delta H(25\;{}^{\circ}C)=-27.36\pm 0.55\;\text{kj}/\text{mole}\\ \;=-6.54\pm 0.13\;\text{kcal}/\text{mole}. \end{array}$$ (4)

The uncertainties in the compositions of the sodium hydroxide solutions and of the samples of (c, II) contributed largely to the overall uncertainties assigned to these equations. In table 4, the corrections for the heat evolved during the solution of (c,I) was made according to eq (4) assuming 0.7 wt percent of (c,I) was present in the samples. This was the average composition found in the samples of table 3. A value comparable to eq (4) at 40 °C is given in group 3, table 6.

The equations representing the reactions of orthoboric acid which were used with eqs (1), (2), and (4) in calculating our best values for the heats of formation of the metaboric acids given in table 8 are

$$\begin{array}{l} H_{3}{\text{BO}}_{3}(c)+1400\;H_{2}O(\text{liq})\to H_{3}{\text{BO}}_{3}(1400\;H_{2}O)\\ \;\Delta H(25\;{}^{\circ}C)=21.60\pm 0.22\;\text{kj}/\text{mole}\\ \;=5.16\pm 0.05\;\text{kcal}/\text{mole} \end{array}$$ (5)

and

$$\begin{array}{l} H_{3}{\text{BO}}_{3}(c)+\text{NaOH}(\text{aq})\to [{\text{NaBO}}_{2}+2H_{2}O](\text{soln})\\ \;\Delta H(25\;{}^{\circ}C)=-22.90\pm 0.34\;\text{kj}/\text{mole}\\ \;=-5.47\pm 0.08\;\text{kcal}/\text{mole}. \end{array}$$ (6)

The average values of the experimental data for these reactions are given in groups 9 and 5 of table 6.

### 5.1. Uncertainties

The estimated error in the analyses of the products by titration was about 0.3 percent. An uncertainty in composition and weight of samples was considered because the samples could not be quantitatively analyzed as to crystal form. This uncertainty was highest in the samples of (c,II) or solution in sodium hydroxide where it was difficult to determine accurately the amount of (c,I) impurity present initially or that which dissolved during the experiment. A greater uncertainty was present in the experiments where the products were not analyzed by titration and the composition of the sample was assumed to be the same as that of another series of experiments. An uncertainty was also considered for the solution of orthoboric acid samples in water in series I where the samples were enclosed in glass bulbs, because some decomposition may have occurred while the samples were under vacuum or during the sealing of the bulbs.

In section 3.3 the failure to reach thermal equilibrium in the calorimeter was discussed and possible errors of 2.0 percent were considered in extreme cases.

It was assumed that the uncertainty in the energy for breaking the platinum disks used in series III did not exceed 4 j/expt. This assumption is based on the work described in section 3.2 where the energies for breaking the disks were determined when the capsule contained water, NaCl, and KCl.

For the experiments in Series I it had been assumed that the variations in the calorimetric sodium hydroxide solutions could be neglected. However, the experiments of Series III were arranged so as to expose any significant effects caused by the variations in the sodium hydroxide solutions on the heats of reaction and to nullify that effect on the heats of formation. These variations were caused by (1) slightly different compositions in the 10*N* stock solutions, (2) changes in the composition of a given stock solution over a period of time, and (3) errors in the amounts weighed during the preparation of the calorimetric solutions. The method of preparing the calorimetric solutions and the analyses of the stock solutions are given in sections 3.1 and 3.4. All of the heats of reaction in sodium hydroxide at 25° C of series III are given in table 7. Two stock solutions, A and B, were used alternately in the preparation of the calorimetric solutions for these experiments, and the experiments are arranged in the table in the chronological order in which they were run. The difference between the results obtained with the two stock solutions is small and within the limits of experimental error. However, a change in composition of the stock solutions with time is indicated by the fact that the results of the earlier experiments with H_3_BO_3_ and (c,III) have a higher mean heat of reaction than those of the comparable later experiments; we have no reason to suspect a change in the composition of the sample. Based on these results we have assigned an uncertainty of 3 percent for the reaction of (c,III) and 1 percent for all other reactions in sodium hydroxide solutions.

Although the overall uncertainties are large for the measured heats of reaction, most of them tend to cancel out in the calculatons of the heats of formation and transition.

## 6. Discussion and Summary

The heats of formation calculated from the various measured heats of reaction are summarized in table 8. Δ*H_r_* was derived from the measured heats of reaction and is represented by the general equation

$${\text{HBO}}_{2}(c,X)+H_{2}\text{O(liq)}\to H_{3}{\text{BO}}_{3}(c).$$

From this, the heats of formation of the metaboric acids were obtained using the following heats of formation at 25 °C: −262.16 kcal/mole for H_3_BO_3_(c) [11, 28], and −68.317 kcal/mole for H_2_O(liq) [17]. The uncertainties given in table 8 were calculated as the square root of the sum of the squares of the individual uncertainties; the largest uncertainty was ±0.32 kcal/mole in the heat of formation of orthoboric acid. We have selected the following as the best values for the heats of formation of the metaboric acids at 25 °C: (c,I), −192.77±0.35 kcal/mole; (c,II), −190.43±0.34 kcal/mole; and (c,III), −189.13±0.34 kcal/mole. These values agreed with the respective values of −192.6, −190.6, and −189.0 kcal/mole obtained by Prosen [18] from measurements of the equilibrium decomposition pressures of orthoboric acid into water vapor and the three metaboric acids.

The best values for the heats of transition were obtained from eq (1) and eq (2),

$$\begin{array}{l} {\text{HBO}}_{2}(c,\text{III})\to {\text{HBO}}_{2}(c,\text{II}),\\ \;\Delta H(25\;{}^{\circ}C)=-1.30\pm 0.05\;\text{kcal}/\text{mole}, \end{array}$$

and from eq (3) and eq (4)

$$\begin{array}{l} {\text{HBO}}_{2}(c,\text{II})\to {\text{HBO}}_{2}(c,I),\\ \;\Delta H(25\;{}^{\circ}C)=-2.33\pm 0.23\;\text{kcal}/\text{mole}. \end{array}$$

Combination of these two values gives the following heat of transition:

$$\begin{array}{l} {\text{HBO}}_{2}(c,\text{III})\to {\text{HBO}}_{2}(c,I),\\ \;\Delta H(25\;{}^{\circ}C)=-3.63\pm 0.24\;\text{kcal}/\text{mole}. \end{array}$$

Roth, Börger, and Bertram [3] measured the heat of solution of a mixture of metaboric acid and orthoboric acid in 0.1*N* sodium hydroxide solution at 19 °C. The crystalline form of the metaboric acid used was not identified; however, they state that the hydration and solution of the metaboric acid may not have been complete in the experiments. This would suggest that their sample contained the monoclinic form which reacts more slowly than the orthorhombic form and usually contains (c,I). They calculate a heat of solution for the metaboric acid, Δ*H*(19 °C) = −8.0 kcal/mole, which is comparable to our value for the monoclinic form given in eq (3).

In the work of von Stackelberg, Quatrain, and Dressel [5], the heat of solution in water was measured at a dilution of 500 H_2_O/H_3_BO_3_ at 20 °C for a mixture of orthoboric acid and metaboric acid. Although their metaboric acid was crystallographically identified as the monoclinic form, they obtained a heat of solution (after correcting for the orthoboric acid) of 0.56 kcal/mole which is near the value we obtained in eq (1) for the orthorhombic form.

The more recent work of Sokolova and coworkers [6] yielded the following values for the heats of solution at 20 °C at a final dilution of 500 H_2_O/H_3_BO_3_: Δ*H* = 0.47 kcal/mole for the orthorhombic, HBO_2_(c,III); and Δ*H*=1.76 kcal/mole for the monoclinic, HBO_2_(c,II). These values are in good agreement with our eq (1) and eq (2). Their heat of transition from the orthorhombic to the monoclinic metaboric acid is −1.29 kcal/mole which agrees with our value of −1.30 kcal/mole.

## Figures and Tables

**Table 1:** Densities of boric and metaboric acid crystals, and liquids used for flotation

Substance	Density
	
	*g*/*cm*^3^
H_3_BO_3_(c)	1.44
Carbon tetrachloride	1.60
HBO_2_(c,III)	1.78
Ethyl Iodide	1.92
HBO_2_(c,II)	2.04
Ethylene bromide	2.17
HBO_2_(c,I)	2.49

**Table 2:** *HBO_2_*(c,III) in water at 25 °C—Series III

Expt. No.	*S*	H_3_BO_3_ total titr.	H_3_BO_3_ ratio	H_3_BO_3_ calc.	HBO_2_ (c, III) calc.	*E_a_*	Δ*Rc*	*Q*	*q*_H3BO3_	Δ*H*(*25* °C)^1^
										
	*g*	*Mole*		*Mole*	*Mole*	*j*/*obm*	*Ohm*	*j*	*j*	*kj*/*mole* HBO_2_(c,III)
1	0.83071	0.01824	0.9623	0.001747	0.01649	21,313.7	−0.002740	−58.40	−38.0	1.662
2	.64787	.01425	.9640	.001303	.01295	21,304.2	− .002212	−47.12	−28.3	1.994
3	.73979	.01621	.9604	.001637	.01457	21,315.3	−.002643	−56.34	−35.6	1.904
4	.66889	.01464	.9582	.001520	.01312	21,300.4	− .002361	−50.29	−33.1	1.844
5	.81347	.01788	.9633	.001666	.01621	21,319.8	− .002845	−60.65	−36.2	1.940
	
Mean										1.869
Standard deviation of the mean										±0.057
Overall uncertainty, 10%										±0.19

1 Another correction, *q_d_*= −7.0 j/expt., was added to *Q.*

**Table 3:** *HBO_2_*(c,II) in water at 25 °C—Series III

Expt. No.	*S*	Undissolved Residue HBO_2_(c,I) titr.	HBO_2_ (c,II) titr.	H_3_BO_3_ ratio	*E_a_*	Δ*Rc*	Q	Δ*H*(25 °C)^1^
								
	*g*	*Mole*	*Mole*		*j*/*ohm*	*Ohm*	*j*	*kj*/*mole* HBO_2_ (c,II) titr.
1	1.11827	0.00016	0.02523	0.9951	21,314.1	−0.008501	−181.19	7.459
2	1.15561	.00018	.02615	.9986	21,303.6	−.008403	−179.01	7.113
3	1.19328	.00008	.02713	.9994	21,318.3	−.009094	−193.87	7.404
4	1.24525	.00019	.02816	.9978	21,322.9	−.009263	−197.51	7.262
5	1.20517	.00026	.02719	.9983	21,286.4	−.009069	−193.05	7.357
6	1.24061	.00020	.02802	.9970	21,302.6	−.009407	−200.39	7.401
	
Mean								7.333
Standard deviation of the mean								±0.051
Overall uncertainty, 3%								±0.220

1 A correction, *q_d_*= −7.0 j/expt., was added to *Q.*

**Table 4:** *HBO_2_*(c,II) in *2N* sodium hydroxide solution at 25 °C—Series III

Expt. No.	*S*	HBO_2_(c,I) calc.	HBO_2_(c,II) calc.	*E_a_*	Δ*Rc*	*Q*	*q*_(c,I)_	Δ*H*(25 °C)^1^
								
	*g*	*Mole*	*Mole*	*j*/*ohm*	*Ohm*	*j*	*j*	*kj*/*mole* HBO_2_(c,II)
1	1.08860	0.000174	0.024670	21,175.9	0.043611	923.50	4.8	−36.956
2	1.23190	.000197	.027918	21,090.1	.049811	1050.52	5.4	−37.185
3	1.32026	.000211	.029920	21,133.0	.053130	1122.80	5.8	−37.099
4	1.39278	.000222	.031563	21,086.4	.056267	1186.47	6.1	−37.175
	
Mean								−37.104
Standard deviation of the mean								±0.053
Overall uncertainty, 2%								±0.74

1 Another correction, *q_d_*= −7.0 j/expt. was added to *Q.*

**Table 5:** *HBO_2_*(c,I) in *2N* sodium hydroxide solution at 25 °C—Series III

Expt. No.	*S*	*E_a_*	Δ*Rc*	*Q*	Δ*H*(25 °C)^1^
					
	*g*	*j*/*ohm*	*Ohm*	*j*	*kj*/*mole* HBO_2_(c,I)
1	0.54449	21,067.7	0.016479	347.17	−27.375
2	.26108	21,073.9	.008065	169.96	−27.350
	
Mean					−27.362
Overall uncertainty, 2%					±0.55

1 A correction, *q_d_*= −7.0 j/expt., was added to *Q.*

**Table 6:** Average results of other solution experiments

Group	Compound	Series	*S*^1^	Undissolved residue HBO_2_(c,I) titr.	Calc. HBO_2_(c,II) or HBO_2_(c,III)	H_3_BO_3_ titr.	*E_a_*	Δ*Rc*	*Q*	*q* (c, I) or *q*_H3BO3_	Dilution H_2_O/H_3_BO_3_	Experimental sdm	ΔH(40 °C)±uncertainty
													
			*g*	*g*	*Mole*	*Mole*	*j*/*ohm*	*Ohm*	*j*	*j*		*kj*/*mole*	$\{\begin{array}{l} kj/mole\\ kcal/mole \end{array}$

Solutions in *2N* sodium hydroxide at 40 °C													

1	H_3_BO_3_(c)	I	1.64442 (1.47–1.76)	---------	--------	-------	23,238.7	0.024556	570.65	--------	--------	±0.007	$\{\begin{array}{r} -21.46\pm 0.21\\ -5.13\pm 0.05 \end{array}$
2	HBO_2_(c,II)	I	1.45515 (1.11–1.91)	0.0359	--------	-------	23,222.7	.049235	1,143.37	32.2	---------	±.077	$\{\begin{array}{r} -35.61\pm 1.78\\ -8.51\pm 0.43 \end{array}$
3	HBO_2_(c,I)	I	0.37354 (0.33–0.42)	-------	--------	--------	23,143.7	.010105	233.85	-------	-------	±.021	$\{\begin{array}{r} -27.43\pm 0.27\\ -6.56\pm 0.07 \end{array}$

Solutions in 2*N* sodium hydroxide at 25 °C													

													Δ*H*(*25* °C)±*uncertainty*
4	H_3_BO_3_(c)	I	0.59862 (0.57–0.62)	---------	--------	--------	22,859.7	.009924	226.86	------	-------	±0.071	$\{\begin{array}{r} -23.43\pm 0.23\\ -5.60\pm 0.06 \end{array}$
5	H_3_BO_3_(c)	III	0.97140 (0.95–1.00)	-------	-------	-------	21,129.8	.017354	366.68	(2)	-------	±.044	$\{\begin{array}{r} -22.90\pm 0.34\\ -5.47\pm 0.08 \end{array}$
6	HBO_2_(c,II)	I	1.34726 (1.19–1.52)	0.0472	--------	--------	22,884.8	.047484	1,086.60	21.2	-------	±.132	$\{\begin{array}{r} -36.87\pm 1.84\\ -8.81\pm 0.44 \end{array}$
7	HBO_2_(c,III)	III	0.69948 (0.66–0.74)	----------	0.013861	--------	21,129.4	.029520	623.75	2 33.8	-------	±.62	$\{\begin{array}{r} -42.07\pm 2.10\\ -10.05\pm 0.50 \end{array}$

Solutions in water at 25 °C													

8	H_3_BO_3_(c)	II	1.57140 (1.38–1.70)	-------	--------	0.02530	21,000.1	−0.026502	−556.55	-------	1000	±0.088	$\{\begin{array}{r} {\;}^{3}22.00\pm 0.22\\ 5.26\pm 0.05 \end{array}$
9	H_3_BO_3_(c)	III	1.10529 (1.06–1.14)	--------	--------	0.01784	21, 276.1	−.017780	−378.28	(2)	1400	±.024	$\{\begin{array}{r} {\;}^{3}21.60\pm 0.22\\ 5.16\pm 0.05 \end{array}$
10	HBO_2_(c,II)	I	1.12472 (1.10–1.17)	0.0647	0.024192	-------	23,122.8	−.007262	−167.91	-------	1000	±.057	$\{\begin{array}{r} 6.94\pm 0.35\\ 1.659\pm 0.083 \end{array}$

1 The range of sample weights is given in parentheses below the average sample weight.

2 A correction, q_d_ = −7.0 j/expt., was also added in the experiments of Series III.

3 Based on moles H_3_BO_3_ titr.

**Table 7:** Table showing the possible effects of time and different concentrations of sodium hydroxide solutions on the measured heats of reaction for experiments in Series III

Sample No.		−Δ*H*(25 °C)		
		NaOH stock soln^1^		Mean
		A	B	
				
		*kj*/*mole*	*kj/mole*	*kj/mole*
H_3_BO_3_(c)	1		23.032	
	2	22.935		22.985
	3		22.987	
HBO_3_(c,III)	1	43.08		
	2		44.22	43.33
	3	42.71		
HBO_3_(c,II)	1	36.956		
	2		37.185	37.104
	3	37.099		
	4		37.175	
HBO_2_(c,I)	1		27.375	
	2	27.350		27.362
HBO_2_(c,III)	4		40.46	
	5	40.51		40.80
	6		41.42	
H_3_BO_3_(c)	4	22.832		
	5		22.836	22.806
	6	22.751		

1 Initial analysis of a calorimetric solution prepared from NaOH stock soln A indicated a normality of 1.79, and from stock soln B, 1.96; no change was indicated in similar analyses made 4 weeks later at the end of these experiments.

**Table 8:** Heats of formation of the metaboric acids as calculated from various reactions (*kcal/mole*)

Compound	Calorimetric solution	Δ*H*(40 °C) for HBO_2_	Δ*H*(40 °C) for H_3_BO_3_	*H_r_*(40 °C)	Δ*H*(25 °C) for HBO_2_	Δ*H*(25 °C) for H_3_BO_3_	Δ*H_r_*(25 °C)	Δ*Hf*°(25 °C)
								
HBO_2_(c,I)	NaOH	--------	-----------	-----------	−6.54	−5.47	−1.07	−192.77±0.35
	NaOH	−6.56	−5.13	−1.43	---------	---------	1 −1.28	−192.56±0.33
HBO_2_(c,II)	NaOH	---------	---------	---------	−8.87	−5.47	−3.40	−190.44±0.38
	NaOH	---------	---------	---------	−8.81	−5.60	−3.21	2 −190.63±0.55
	NaOH	−8.51	−5.13	−3.38	---------	---------	1 −3.23	2 −190.61±0.55
	H_2_O	---------	---------	---------	1.66	5.26	−3.60	2 −190.24±0.35
	H_2_O	---------	---------	---------	1.75	5.16	−3.41	−190.43±0.34
HBO_2_(c,III)	H_2_O	---------	---------	---------	0.45	5.16	−4.71	−189.13±0.34
	NaOH	---------	---------	---------	−10.05	−5.47	−4.58	2 −189.26±0.61

1 Estimated thermal coefficient, Δ*Cp*~—10 cal/deg mole.

2 These values are subject to large uncertainties in the composition of the samples.

## Footnotes

1 Figures in brackets indicate the literature references at the end of this paper.

2 A. Perloff of the Constitution and Microstructure Section of the Minera Products Division.

3 C. P. Saylor of the Pure Substances Section of the Analytical and Inorganic Chemistry Division.

4 Spectrochemistry Section of the Analytical and Inorganic Chemistry Division.
